# Cholesterol-mediated allosteric regulation of the mitochondrial translocator protein structure

**DOI:** 10.1038/ncomms14893

**Published:** 2017-03-30

**Authors:** Garima Jaipuria, Andrei Leonov, Karin Giller, Suresh Kumar Vasa, Łukasz Jaremko, Mariusz Jaremko, Rasmus Linser, Stefan Becker, Markus Zweckstetter

**Affiliations:** 1 Deutsches Zentrum für Neurodegenerative Erkrankungen (DZNE), Von-Siebold-Strasse 3a, 37075 Göttingen, Germany; 2 Max-Planck-Institut für Biophysikalische Chemie, Am Fassberg 11, 37077 Göttingen, Germany; 3 Department of Neurology, University Medical Center Göttingen, University of Göttingen, Waldweg 33, 37073 Göttingen, Germany

## Abstract

Cholesterol is an important regulator of membrane protein function. However, the exact mechanisms involved in this process are still not fully understood. Here we study how the tertiary and quaternary structure of the mitochondrial translocator protein TSPO, which binds cholesterol with nanomolar affinity, is affected by this sterol. Residue-specific analysis of TSPO by solid-state NMR spectroscopy reveals a dynamic monomer–dimer equilibrium of TSPO in the membrane. Binding of cholesterol to TSPO's cholesterol-recognition motif leads to structural changes across the protein that shifts the dynamic equilibrium towards the translocator monomer. Consistent with an allosteric mechanism, a mutation within the oligomerization interface perturbs transmembrane regions located up to 35 Å away from the interface, reaching TSPO's cholesterol-binding motif. The lower structural stability of the intervening transmembrane regions provides a mechanistic basis for signal transmission. Our study thus reveals an allosteric signal pathway that connects membrane protein tertiary and quaternary structure with cholesterol binding.

## Abstract (web summary)

The outer mitochondrial membrane translocator protein (TSPO) mediates several mitochondrial functions and binds cholesterol with a high affinity. Here the authors use solid-state NMR to show that cholesterol binding to TSPO results in allosteric changes that modulate TSPO oligomerization.

Cholesterol regulates the structure and function of membrane proteins^1^,^2^. The involved mechanisms, however, are poorly understood. This is because of the variability and complexity of membranes, the range of conformations the cholesterol molecule can adopt and the number of possible interactions between proteins, cholesterol and membrane lipids, as well as technical challenges associated with the dynamic nature of protein–lipid interactions^3^,^4^. A powerful method to overcome these challenges might be solid-state nuclear magnetic resonance (NMR), which has recently emerged as a viable technology for the study of complex insoluble biomolecules at atomic resolution^3^,^5^,^6^,^7^. Indeed, due to the possibility to perform solid-state NMR measurements of proteins embedded into phospholipid bilayers, unique insights into the influence of lipids on membrane protein structure can be obtained^8^,^9^.

An important membrane protein that is expressed at high levels in the outer mitochondrial membrane of steroidogenic cells of the nervous system is the translocator protein TSPO^10^,^11^,^12^. TSPO is evolutionarily conserved across species ranging from prokaryotes to eukaryotes^13^. The function of the mammalian protein has been related to transport of cholesterol across the mitochondrial membrane, mitochondrial respiration, cell proliferation and apoptosis^10^,^11^,^12^. Binding of cholesterol to TSPO occurs with nanomolar affinity^14^. In addition, cholesterol binding can be inhibited by single point mutations within a specific sequence motif at the carboxy-terminal end of TSPO (residues A147-S159), the so-called cholesterol recognition amino acid consensus (CRAC) motif^14^,^15^.

A crucial attribute of TSPO is its elevated expression in response to a variety of cancers and neurological diseases, such as Alzheimer's disease and Parkinson's disease, as well as psychiatric disorders including depression and anxiety^12^,^16^,^17^. The increased expression of TSPO in these pathological states has been exploited to treat TSPO as a potential clinical biomarker^12^,^17^ and to explore its therapeutic capacities^18^,^19^. This is possible due to TSPO's ability to bind to synthetic ligands with high specificity, which then allows its abnormal expression levels to be traced and potentially treated.

Detailed insight into the structure of TSPO alone and in complex with the diagnostic ligand (R)-1-(2-chlorophenyl)-*N*-methyl-*N*-(1-methylpropyl)-3-isoquinolinecarboxamide (PK11195) was provided by solution-state NMR^20^, and into the structure of bacterial homologous proteins by X-ray crystallography^21^,^22^. The TSPO structure is formed by five transmembrane helices. The transmembrane helices are arranged in such a way that they provide a hydrophobic pocket at the cytosolic side of the mitochondrial membrane, to which radio ligands can bind with high affinity^20^. The cholesterol-binding motif is also located at the cytosolic end of transmembrane helix 5, but the side chains of residues that are essential for binding to cholesterol do not point to the PK11195 binding site, but instead to the hydrophobic membrane environment^20^. Cholesterol binding could thus influence both the tertiary and quaternary structure of TSPO.

Here we investigate the structure of mammalian TSPO embedded into the physiological environment of a membrane. Using solid-state NMR spectroscopy, we demonstrate that binding of cholesterol promotes monomerization of TSPO through an allosteric mechanism that connects the cholesterol-binding motif at the cytosolic end of TSPO with the GxxxG oligomerization motif on the opposite side of the TSPO structure. Through this allosteric mechanism, cholesterol can regulate TSPO homo- and hetero-oligomerization, and thereby influence mitochondrial function.

## Results

### High-resolution solid-state NMR of TSPO

**Figure 1:**
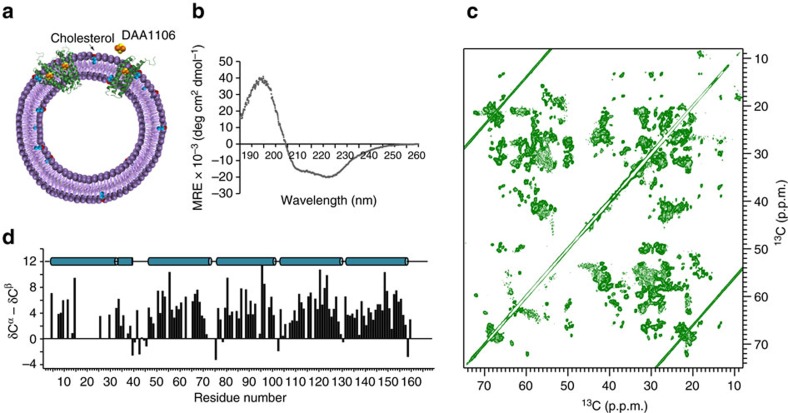
High-resolution solid-state NMR of mammalian TSPO in a lipid bilayer. (**a**) Schematic representation of mammalian TSPO embedded into liposomes. Cholesterol is marked in red and cyan, the TSPO-ligand DAA1106 in orange/yellow. (**b**) CD spectrum of the membrane-embedded mTSPO/DAA1106 complex. (**c**) High-resolution ^13^C–^13^C proton-driven spin diffusion spectrum of mTSPO bound to DAA1106. (**d**) Secondary structure analysis of liposome-embedded mTSPO in complex with DAA1106. Positive δC^α^-δC^β^ values are indicative of α-helix and identify five transmembrane helices and a short cytosolic helix following transmembrane helix I.

To obtain insight into the influence of cholesterol on the structure of TSPO, we reconstituted TSPO from mouse (mTSPO) into liposomes formed by 1,2-dimyristoyl-sn-glycero-3-phosphocholine (DMPC), which was already successfully used for solid-state NMR measurements of membrane proteins, using a molar ratio of 1:20 (Fig. 1a). As mTSPO is flexible in the absence of high-affinity ligands (Supplementary Fig. 1)^23^,^24^, reconstitution into liposomes was performed after binding of the small molecule *N*-(2,5-Dimethoxybenzyl)-*N*-(5-fluoro-2-phenoxyphenyl)acetamide (DAA1106). According to competition studies, DAA1106 binds to the same binding site as the endogenous TSPO ligand porphyrin and the radioligand PK11195 (ref. 25), for which the complex structure with mTSPO has been determined^20^. In agreement with a well-folded helical transmembrane structure (Fig. 1b), two-dimensional nitrogen–carbon and carbon–carbon correlation experiments showed a large number of defined NMR signals (Fig. 1c).

**Figure 2:**
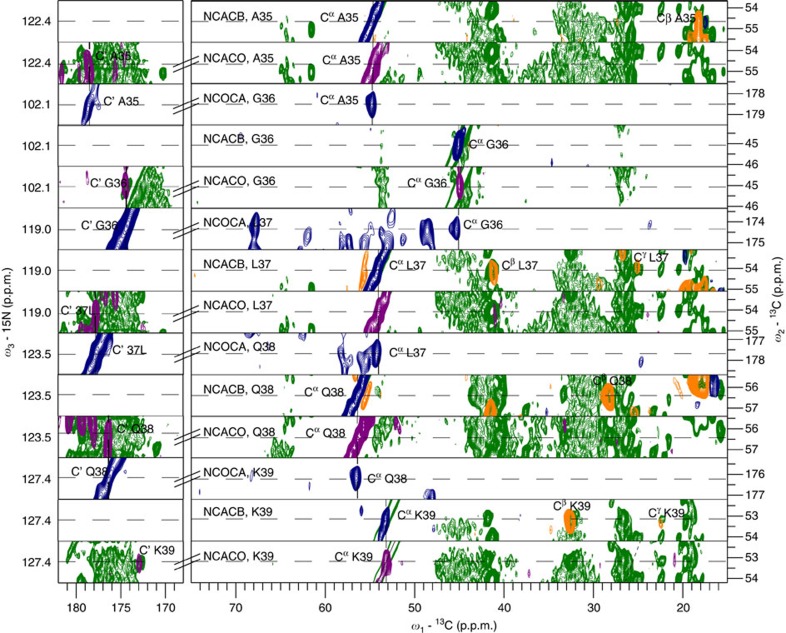
Sequence-specific resonance assignment of membrane-embedded mTSPO (bound to DAA1106). Strip plots of 3D NCACB (diagonal peak—blue, cross peak—orange), 3D NCOCA (cross peak—blue) and NCACO (cross peak—purple) used for sequence-specific resonance assignments. Corresponding regions from the 2D proton-driven spin diffusion (green) are superimposed for comparison.

**Figure 3:**
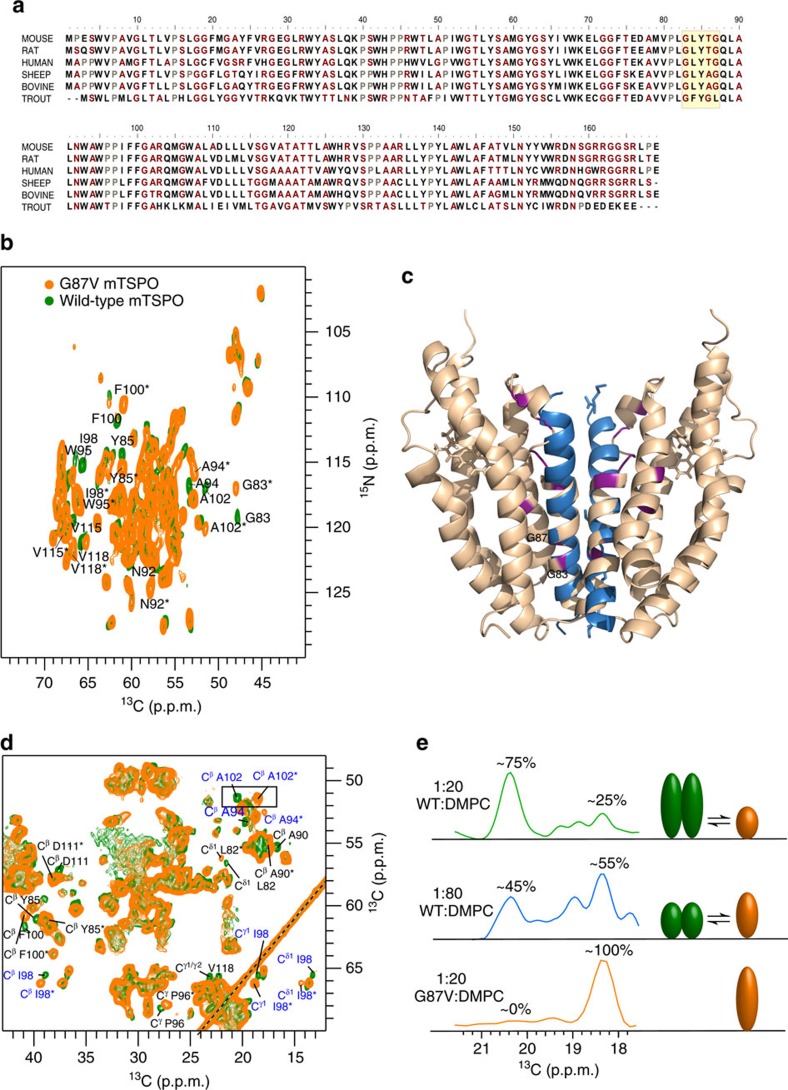
Mammalian TSPO populates a dynamic monomer/dimer equilibrium in the membrane. (**a**) Conservation of the amino acid sequence of mammalian TSPO. The GxxxG motif in transmembrane helix III is highlighted in yellow. (**b**) Superposition of nitrogen-carbon correlation spectra of wild-type mTSPO (green) and its G87V mutant (orange) both in complex with DAA1106. Residues, which show two cross-peaks, are labelled. Peaks corresponding to the mTSPO monomer are marked by stars. (**c**) Model of the mTSPO dimer with an interhelical arrangement as observed in glycophorin A (shown in blue). Residues that show two cross-peaks in **b** are highlighted in purple. (**d**) Superposition of carbon–carbon correlation spectra of wild-type mTSPO/DAA1106 (green) and its G87V variant (orange). The C^α^/C^β^ peaks of A102, which were used for monomer/dimer quantification, are highlighted. Residues with two conformations (two peaks in proton-driven spin diffusion /NCA/NCACB) are marked in blue. Residues with shifted side-chain resonances are labelled in black. (**e**) One-dimensional traces at the C^β^ resonance of A102 in wild-type mTSPO at protein-to-DMPC molar ratios of 1:20 (top) and 1:80 (middle). In case of the G87V-mTSPO, only the monomer peak of A102 was observed (bottom).

To determine the resonance assignment of the 169-residue mTSPO (Fig. 3a), we recorded a set of dedicated solid-state NMR experiments (Fig. 2 and Supplementary Table 1). Given that the quality of the resulting spectra was high (Fig. 1c) and resonance assignments of the mTSPO/PK11195 complex in micelles had previously been obtained^20^,^26^, almost complete backbone assignments of the membrane-embedded protein were achieved. The resonance assignments then allowed characterization of the secondary structure of membrane-embedded mTSPO with single-residue resolution. Indeed, for most residues a positive difference between δC^α^ and δC^β^ (δ represents the deviation from random coil chemical shift values) was observed (Fig. 1d), in agreement with the presence of five transmembrane helices. Bioinformatic work had predicted five transmembrane helices with the C terminus pointing to the cytosol and the structure of the mTSPO/PK11195 complex in micelles showed a tight helical bundle^20^,^27^. Although three mTSPO loops are very short, a cytosolic α-helix follows TM-I^20^ (Fig. 1d).

### A dynamic monomer–dimer equilibrium of TSPO

Detailed analysis of the solid-state NMR spectra revealed two signal sets for several mTSPO residues (Fig. 3b, marked by stars). Most of these residues belong to transmembrane helix TM-III. One of the TM-III residues that showed two well-separated NMR signals in the nitrogen–carbon correlation spectrum is G83 (Fig. 3b,c). Together with G87, G83 forms a GxxxG motif (Fig. 3a), which constitutes a widespread protein–protein interaction element. The presence of the GxxxG motif in TM-III suggests that the duplication of NMR signals was caused by transmembrane oligomerization^28^. Consistent with this hypothesis, TSPO has been found in oligomeric states in cells^29^. Moreover, a bacterial protein from *Bacillus cereus*, which is homologous to mTSPO, crystallized as dimer^22^. The dimer interface of the bacterial protein includes the GxxxG motif and the inter-dimer arrangement is highly similar to that observed for the GxxxG prototype of glycophorin A (Supplementary Fig. 2)^30^. To test the hypothesis that mTSPO dimerizes in the membrane, we mutated G87 into valine. Indeed, for G87V-mTSPO only a single set of NMR resonances was observed (Fig. 3b,d). In addition, the spectral quality improved in multi-dimensional solid-state NMR experiments (Supplementary Table 2), enabling the assignment of further residues in TM-I. Thus, wild-type mTSPO populates a dynamic monomer–dimer equilibrium in the membrane, whereby dimerization of mTSPO depends on the identity of the residue at position 87, suggesting that dimerization is mediated by the ^83^GxxxG^87^ motif in transmembrane helix TM-III. Notably, TSPO proteins from other species might use different mechanisms of oligomerization. For example, some TSPOs from fishes have no G87, but instead L87 (Fig. 3a). In addition, the TSPO paralogous protein TSPO2 forms oligomers^31^, but contains a phenylalanine at position 87 (ref. 13).

As NMR signal intensities depend on the concentration of the contributing species, the presence of distinct signals for monomeric and dimeric mTSPO allowed us to quantify their respective populations in the membrane (Fig. 3d,e). Quantification showed that at a protein-to-lipid molar ratio of 1:20 ∼75% of the mTSPO/DAA1106 complex was present in dimeric form and only 25% as monomer. The monomer–dimer equilibrium depends on the protein-to-lipid ratio, as lowering the ratio to 1:80 decreased the dimer population to ∼45% (Fig. 3e). In case of G87V-mTSPO, only the monomer was observed. Owing to the less-defined structure of membrane-embedded mTSPO in the absence of DAA1106 (Supplementary Fig. 1), we currently cannot quantitatively dissect the monomer/dimer-population of unbound mTSPO, but antibodies detected monomers and dimers also for the ligand-free wild-type protein^29^.

### Dimerisation couples with long-range changes in structure

**Figure 4:**
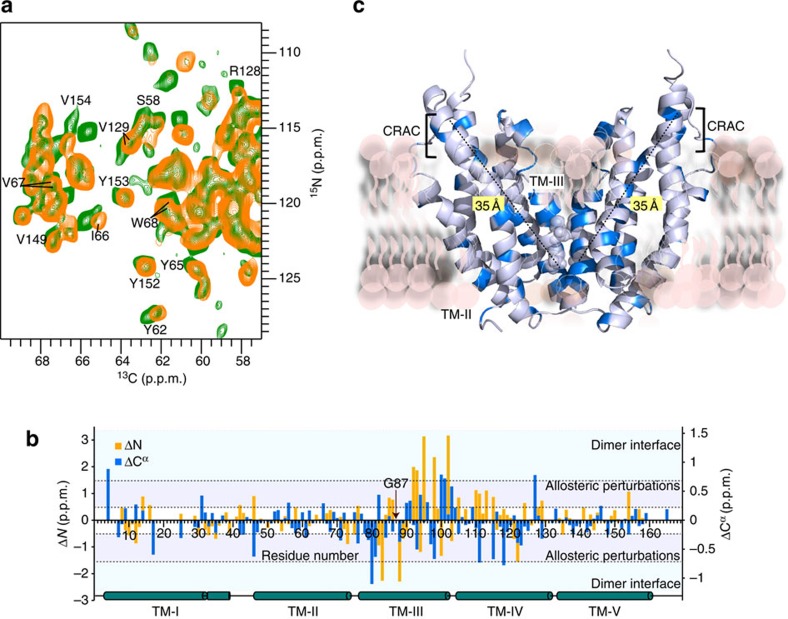
TSPO dimerization is coupled to long-range changes in the tertiary structure of TSPO. (**a**) Residues up to 35 Å away from mTSPO's ^83^GxxxG^87^ oligomerizatin motif experience changes in their chemical environment upon protein monomerization. NCA spectra of wild-type TSPO/DAA1106 and its G87V mutant are shown in green and orange, respectively. Perturbed residues include Y153 and V154 in TSPO's cholesterol-binding motif. (**b**) Residue-specific chemical shift differences between the dimeric and monomeric form of mTSPO (both in complex with DAA1106). Chemical shift perturbations Δ*N*>1.5 p.p.m. and ΔC^α^>0.75 p.p.m. were observed near the dimer interface. Additional chemical shift perturbations (0.5<ΔN<1.5 p.p.m., 0.25<ΔC^α^<0.75 p.p.m.) report on allosteric changes. (**c**) Residues with ^13^C^α^ chemical shift perturbations >0.25 p.p.m. in **b** were mapped in blue colour onto the dimer model of mTSPO. G83 and G87, which form the GxxxG oligomerization motif in TM-III, are represented by spheres.

How does the transition from dimer to monomer affect the structure of the mTSPO/DAA1106 complex in the membrane? To address this question, we analysed the chemical shifts of mTSPO and its monomeric variant G87V-mTSPO (Fig. 4a). The strongest changes in TSPO's signal positions were observed for residues in TM-III (Fig. 4b). They report on changes in the chemical environment and are caused by the G87V-mutation and the associated loss of the dimer interface. In addition, several residues that are not part of TM-III were perturbed, including Y62, I66, V67, W68 and L71 in TM-II, as well as R128/V129 in TM-IV (Fig. 4a). These residues are not in direct vicinity to the mutation site and are not part of the dimer interface (Fig. 4c). The structural perturbations even reach V149, Y152, Y153 and V154 (Fig. 4a). V149, Y152, Y153 and V154 belong to the CRAC motif at the C terminus of mammalian TSPO (Fig. 3a). Mutation of Y152, Y153 and R156 has previously been shown to abolish binding of cholesterol to mTSPO^14^,^15^. Thus, structural differences between the TSPO monomer and dimer occur far outside of the dimerization interface and reach the cholesterol-binding site that is 35 Å away from the ^83^GxxxG^87^ motif in TM-III (Fig. 4c).

### Cholesterol binding influences TSPO oligomerization

**Figure 5:**
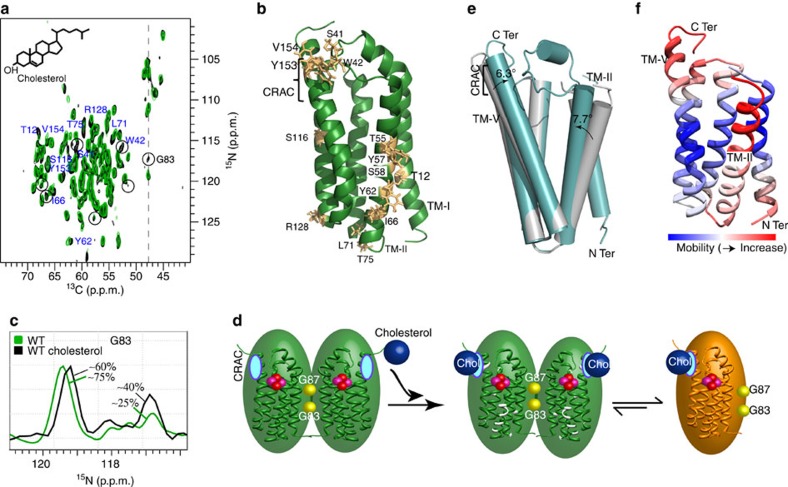
An allosteric signal pathway that connects cholesterol binding to membrane protein tertiary and quaternary structure. (**a**) Comparison of solid-state NMR spectra of mTSPO (bound to DAA1106) in the absence (green) and presence (black) of a tenfold excess of cholesterol. Residues that undergo chemical shift changes are labelled. Circled cross-peaks increase in intensity. (**b**) Residues affected in **a** are highlighted in gold on the 3D structure of mTSPO (PDB id: 2MGY). The cholesterol-binding motif (CRAC) in TM-V is indicated. (**c**) Quantification of the dimer/monomer equilibrium in the absence (green) and presence (black) of cholesterol. Spectral traces at the C^α^ chemical shift of G83 (dashed line in **a**) are shown. (**d**) Schematic representation illustrating the connection between cholesterol binding, allosteric changes in tertiary structure (marked in white) and TSPO dimer dissociation. The CRAC motif before binding of cholesterol is shown in cyan. DAA1106 is shown in a sphere representation (pink/red). In the mTSPO monomer (gold), the ^83^GxxxG^87^ motif in TM-III is available for interaction with other proteins of the outer mitochondrial membrane. (**e**) Crystal structures of bacterial proteins (PDB id: 4UC2 (green) and 4UC3 (grey)), which are homologous to mammalian TSPO, highlight the ability of transmembrane helices TM-V and TM-II to undergo changes in transmembrane orientation. (**f**) Mobility of different regions in the TSPO fold as predicted by Gaussian network analysis. Gaussian network analysis was performed for PDB id 4UC3. The least stable transmembrane parts of the TSPO fold are the CRAC motif in TM-V and the transmembrane helix TM-II.

Cholesterol binds with nanomolar affinity to the CRAC motif at the C terminus of mammalian TSPO^14^. To obtain insight into the influence of cholesterol on the tertiary and quaternary structure of membrane-embedded TSPO, we reconstituted the mTSPO/DAA1106 complex into DMPC liposomes containing a tenfold excess of cholesterol over protein. Subsequently, multi-dimensional solid-state NMR experiments were recorded and compared with those obtained in the absence of cholesterol. In both the presence and absence of cholesterol, the spectra of the mTSPO/DAA1106 complex were highly resolved (Fig. 5a). Moreover, the cross-peaks closely superimposed with changes in signal position localized to specific residues (Fig. 5a and Supplementary Fig. 3). The perturbed residues included Y153 and V154, in agreement with binding of cholesterol to the CRAC motif. The chemical environment was also changed in the vicinity to S41 and W42, which are in direct spatial proximity to the CRAC motif (Fig. 5b). Other residues further away from the CRAC motif were S116 and R128 in TM-IV and T12 in TM-I (Fig. 5a,b). Most of the residues that showed conformational fluctuations upon addition of cholesterol, however, were in TM-II (Fig. 5b). In addition, the N-CA monomer resonance of G83 increased in intensity (Fig. 5a,c). Quantification of NMR intensities showed that in the presence of cholesterol the monomer population increased from ∼25 to ∼40% (Fig. 5c and Supplementary Fig. 3). Thus, binding of cholesterol promotes dissociation of the mTSPO dimer into a monomeric species.

### CRAC is structurally linked to the dimerization interface

Cholesterol increases the rigidity of membranes^32^. The observed perturbations of the structure of the mTSPO/DAA1106 complex, as well as the shift in the monomer–dimer equilibrium, could therefore be caused by changes in the properties of the phospholipid bilayer and not by direct binding of cholesterol to the CRAC motif. Although such an indirect contribution cannot be excluded, the changes observed in the solid-state NMR spectra upon introduction of the G87V mutation support a direct signal transfer pathway connecting the CRAC motif in TM-V with the ^83^GxxxG^87^-dimerization motif in TM-III (Fig. 4). To further support this connection, we substituted Y152 in the CRAC motif by serine. This resulted in an overall decrease in the quality of the solid-state NMR spectra (Supplementary Fig. 4a) recorded under otherwise identical sample conditions. Notably, the spectra were similar in the absence and presence of cholesterol (Supplementary Fig. 4b), in agreement with impaired binding of cholesterol to Y152S-mTSPO^33^. What is, however, the cause for the mutation-induced decrease in spectral quality? In the mTSPO/PK11195 structure^20^, the Y152 side chain points to the hydrophobic environment of the membrane and is not in direct contact with the radioligand, whereas affinity measurements showed a lower affinity of Y152S-mTSPO for radioligands^33^. The amino acid at position 152 might therefore be important for the structural integrity of the CRAC motif, which will influence radioligand binding at its interior side. Consistent with this hypothesis, cross-peaks from several residues in the CRAC motif (T148, V149, Y153, V154, N158) were either not detected or shifted in the spectra of Y152S-mTSPO/DAA1106, whereas well-defined resonances from other parts of the mTSPO structure were present (Supplementary Fig. 4a). In addition, no evidence for a dimeric Y152S-mTSPO species was detected. G83, Y85, I98 and A102, which are sensitive reporters of the oligomerization state of mTSPO (Fig. 3), were all located at the monomer chemical shift (Supplementary Fig. 4a), suggesting that perturbation of the CRAC motif by Y152S is transmitted to mTSPO's dimerization interface.

### A signal transmission model based on structural stability

Although cholesterol binds to the C terminus of TM-V, G87 is located in TM-III about 35 Å from the CRAC motif. Comparison of the chemical shift changes induced by these two distinct perturbations (Figs 4 and 5) identified regions within the mTSPO structure that neither belong to TM-III nor the CRAC motif but sense both modifications. Such chemical shift changes are located in particular in transmembrane region TM-II. Thus, TM-II could be the switch that relays the signal from one side of the protein to the other side and is critical for the cholesterol-induced change in the monomer–dimer equilibrium of membrane-embedded mTSPO (Fig. 5d). How might the signal transfer be structurally encoded? Comparison of carbon chemical shifts observed for the dimeric and monomeric species of the mTSPO/DAA1106 complex showed that in both states the local structure of mTSPO's five transmembrane helices is only little perturbed (Supplementary Fig. 5). Thus, structural changes required for signal transfer might not be related to perturbations of the TM helices, but rather made possible through rearrangements in their relative orientation. Such a signal transfer model is supported by changes that were observed between the wild-type TspO protein from *Rhodobacter sphaeroides* and its A138T mutant^21^. The crystal structures of these two proteins were highly similar, with the exception of a change in the orientation of TM-V by 6.3° and TM-II by 7.7° (Fig. 5e). Further support for the importance of TM-II for allosteric modulation comes from an analysis of the stability of different regions of the TSPO structure. Application of a Gaussian network analysis^34^,^35^ to the available three-dimensional (3D) structures of TSPO and its bacterial homologues consistently identified the CRAC motif in TM-V, the neighbouring TM-III/TM-IV loop and the transmembrane helix TM-II as the least stable parts of the TSPO fold (Fig. 5f and Supplementary Fig. 6). Thus, a signal transfer pathway appears that takes advantage of structurally less restricted transmembrane regions.

Our study reveals an allosteric signal pathway that connects membrane protein tertiary and quaternary structure with cholesterol binding. As binding of cholesterol to a site distinct from the dimer interface promotes monomer formation of mTSPO, an increased fraction of mTSPO molecules will have a free GxxxG motif at higher concentrations of cholesterol. In contrast, an increase in TSPO expression during disease will increase the protein-to-lipid ratio and thereby favor oligomerization of mTSPO. Depending on the lipid composition of the membrane, the concentration of cholesterol and the local tissue-specific protein concentration, the GxxxG motif of mouse/human TSPO (Fig. 3a) will be available to interact with other proteins in the outer mitochondrial membrane^10^,^12^ and thereby influence mitochondrial function^29^,^36^.

## Methods

### Protein preparation

Mouse TSPO was expressed in *Escherichia coli* in M9 minimal medium with ^13^C_6_-D-glucose as the carbon source and ^15^N-NH_4_Cl as the nitrogen source^20^,^37^. The protein was solubilized from inclusion bodies with buffer A (150 mM NaCl, 50 mM Hepes pH 7.8, 1% (w/v) SDS). After loading onto a Ni-NTA column, the detergent was switched to 2% (m/v) dodecylphosphocholine (DPC) on column. The protein was eluted in DPC with 250 mM imidazole. For reconstitution into liposomes^38^, either loaded with DAA1106 or unloaded, the mTSPO protein in DPC micelles was incubated with DMPC liposomes at a specific protein/lipid ratio for 2 h at room temperature. After removal of the detergent with biobeads (BioRad), liposomes were pelleted by centrifugation at 125,000 *g*. The liposome pellet was washed with 10 mM sodium phosphate pH 6.0, pelleted and transferred into an NMR rotor.

### NMR spectroscopy

NMR samples contained ∼12–15 mg of ^13^C/^15^N-labelled protein packed in a 3.2 mm rotor with DSS added externally for temperature calibration. Experiments were recorded either on a 950 MHz Bruker Avance III HD standard-bore spectrometer equipped with a 3.2 mm (^1^H, ^13^C and ^15^N) *E*_free_ triple resonance probe (Bruker Biospin) or on a 850 MHz wide-bore spectrometer equipped with 3.2 mm triple resonance probe (Bruker Biospin). All solid-state NMR experiments were recorded at 5 °C. 90° pulse widths of 2.5 μs for ^1^H, 4.5 μs for ^13^C, 6.7 μs for ^15^N and ^1^H decoupling strengths of 80–100 kHz were used. Acquisition parameters and measurement times for two-dimensional (2D) ^13^C–^13^C proton-driven spin diffusion spectra^39^,^40^, 2D NCA, 2D NCO, 3D NCACB, 3D NCACX, 3D NCOCX, 3D NCOCA, 3D NCACO, 3D CANCO are provided below. Spectra were processed in Topspin (Bruker) and analysed with CcpNmr-Analysis^41^ and CARA^42^. Sequential resonance assignment of liposome-embedded mTSPO was achieved following established solid-state NMR approaches^5^,^6^,^43^,^44^ and was further helped by the assignment of the mTSPO/PK11195-complex solubilized in detergent micelles^20^,^26^.

### Gaussian network analysis

The flexibility of different regions of the TSPO fold was calculated based on the available 3D structures of TSPO and its bacterial homologues using a standard Cα-atom based Gaussian Network Model available as online server oGNM^45^. The used structures were: mTSPO (PDB id: 2MGY), TspO from *B. cereus* (PDB id: 4RYJ), wild-type TspO from *R. sphaeroides* (PDB id: 4UC3) and A138T-TspO from *R. sphaeroides* (PDB id: 4UC2). Only the monomeric subunits were used, to obtain insight into the intrinsic stability of the arrangement of the five transmembrane helices.

### Data availability

Data supporting the findings of this study are available within the article and its Supplementary Information files and from the corresponding author upon reasonable request. The PDB structures with accession codes 4RYJ, 5EH4, 2MGY, 4UC2 and 4UC3 were used in this work.

## Additional information

**How to cite this article:** Jaipuria, G. *et al*. Cholesterol-mediated allosteric regulation of the mitochondrial translocator protein structure. *Nat. Commun.* **8,** 14893 doi: 10.1038/ncomms14893 (2017).

**Publisher's note**: Springer Nature remains neutral with regard to jurisdictional claims in published maps and institutional affiliations.

## Supplementary Material

Supplementary Information Supplementary Figures, Supplementary Tables, Supplementary Methods, Supplementary References.
