# Supplementary material for: Cholesterol-mediated allosteric regulation of the mitochondrial translocator protein structure
Source: Nat Commun. 2017 Mar 30;8:14893. doi: 10.1038/ncomms14893 (PMC5379104; doi:10.1038/ncomms14893)
Supplement: Supplementary Information — Supplementary Figures, Supplementary Tables, Supplementary Methods, Supplementary References. [file ncomms14893-s1.pdf]

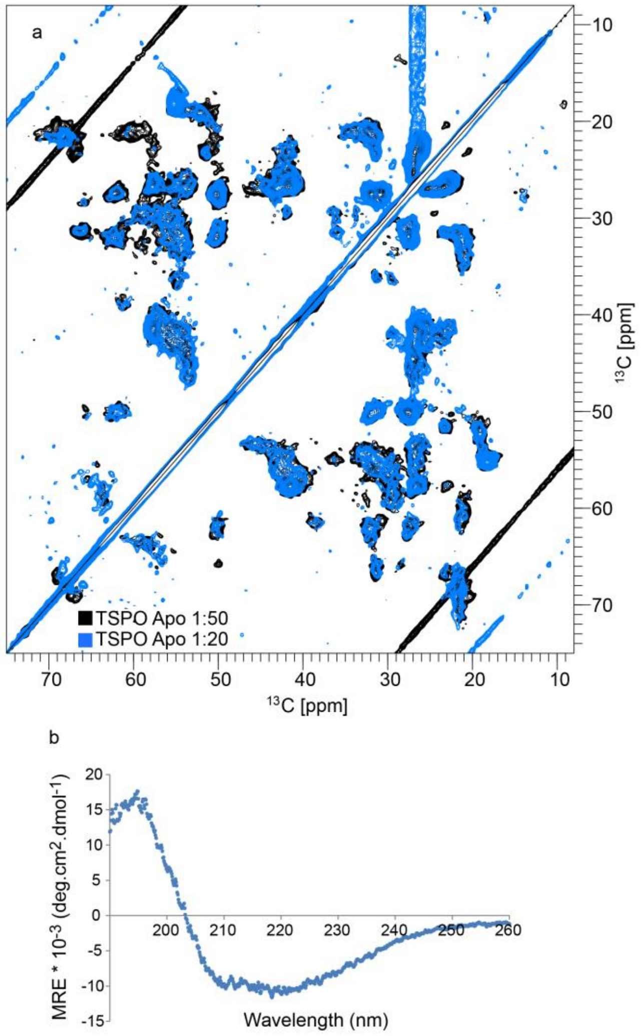

Supplementary Figure 1 | Membrane-embedded mTSPO in the absence of DAA1106. (**a**) Solid-state ^{13}C-^{13}C PDSD spectra of apo mTSPO reconstituted into DMPC liposomes. Protein-to-lipid molar ratios of 1:20 (blue; recorded at 800 MHz) and 1:50 (black; recorded at 950 MHz) are shown. (**b**) Far-UV circular dichroism spectrum of the 1:20 sample in (a).

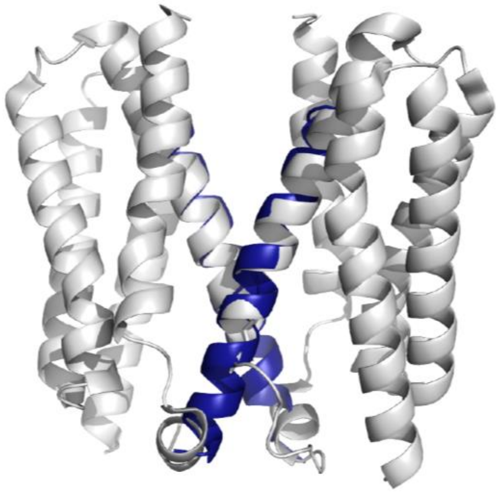

Supplementary Figure 2 | The GxxxG motif in bacterial TspO. Superposition of the dimeric crystal structure of TspO from *Bacillus cereus* (grey; PDB id: 4RYJ) with the canonical GxxxG inter-helix arrangement of glycophorin A (blue; PDB id: 5EH4). The relative orientation between the two helices, which contain the GxxxG motif and thereby form the dimerization interface, is highly similar.

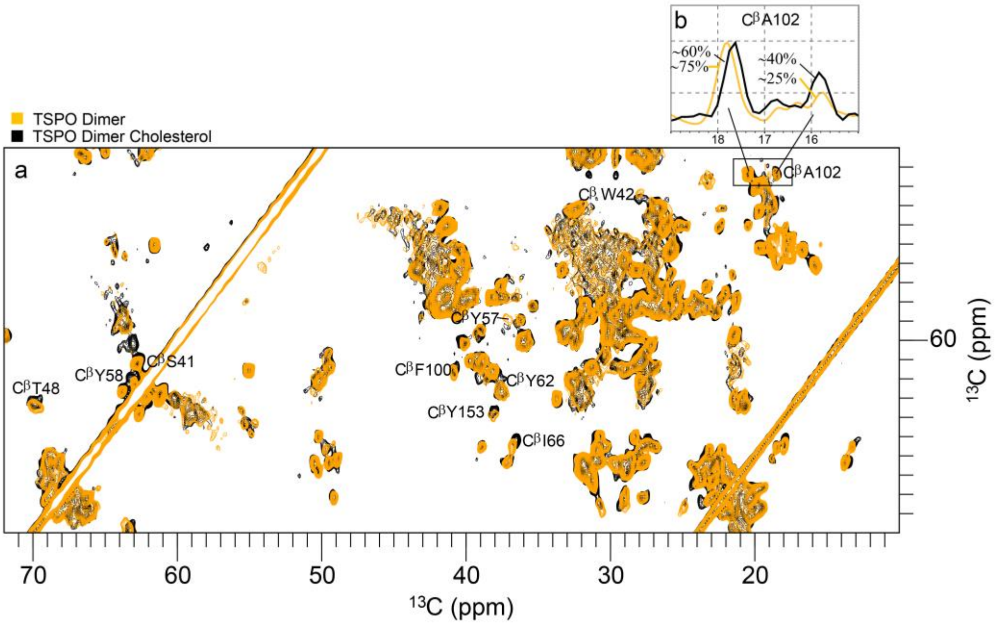

Supplementary Figure 3 | Cholesterol causes allosteric structural changes and promotes dissociation of the mTSPO dimer. (**a**) Superposition of 2D ^{13}C-^{13}C PDSD spectra of mTSPO (bound to DAA1106) in the absence (gold) and presence (black) of a 10-fold excess of cholesterol. The mixing time was 20 ms. Residues, which underwent chemical shift changes, are labelled. (**b**) 1D traces for C^{β} A102 taken from PDSD spectra in the absence (gold) and presence (black) of cholesterol.

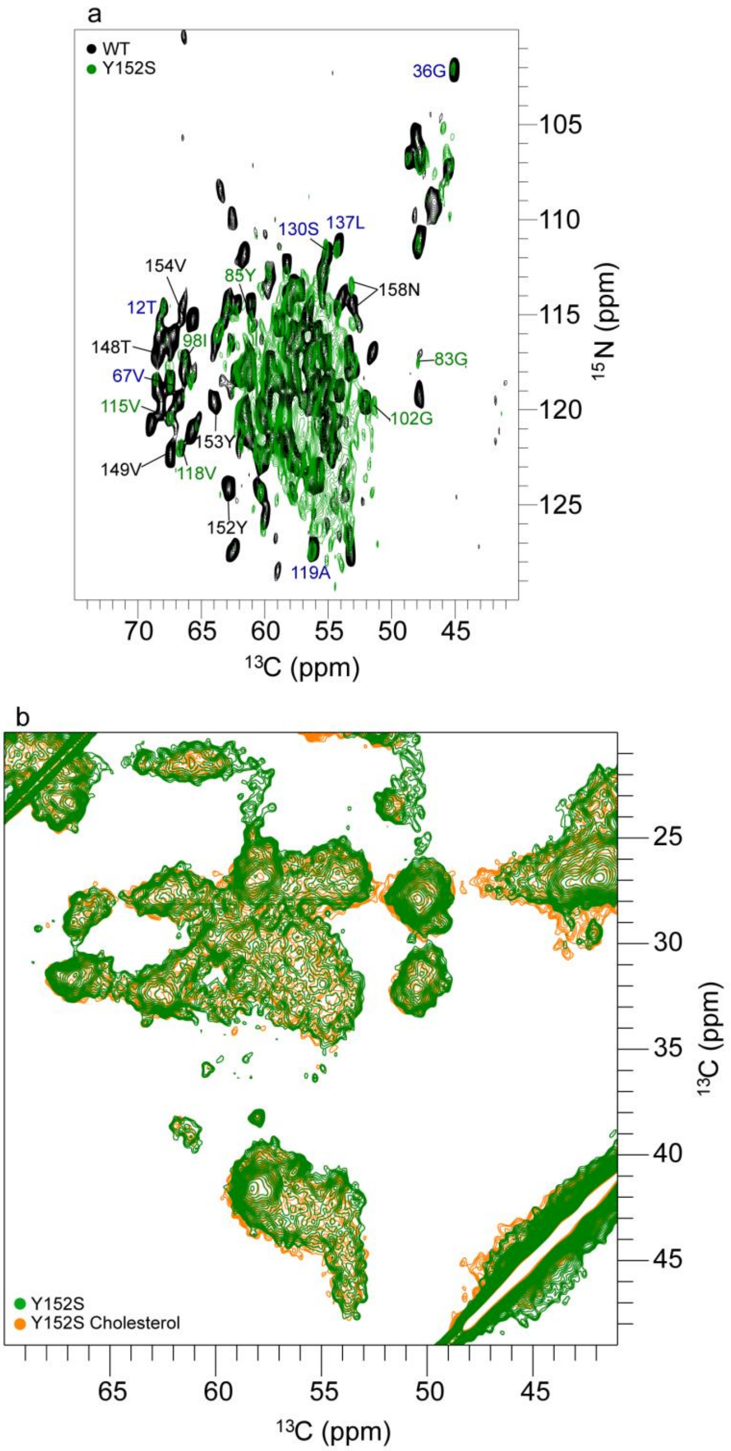

Supplementary Figure 4 | Solid-state NMR spectra of Y152S-mTSPO (green) reconstituted into DMPC liposomes. Before reconstitution the protein was loaded with DAA1106 following the same procedure as for the wild-type protein. (**a**) 2D ^{15}N-^{13}C$^{\alpha}$ spectrum. The spectrum of wild-type mTSPO in complex with DAA1106 is shown in black for comparison. Cross-peaks are labelled by their residue name: disappearing peaks in black, largely unperturbed peaks in blue and residues, which appear at the monomer chemical shift in Y152S-mTSPO, in green. (**b**) Superposition of a selected region from a ^{13}C-^{13}C PDSD spectrum of Y152S-mTSPO (with DAA1106) in the absence (green) and presence (orange) of a 10-fold excess of cholesterol over protein.

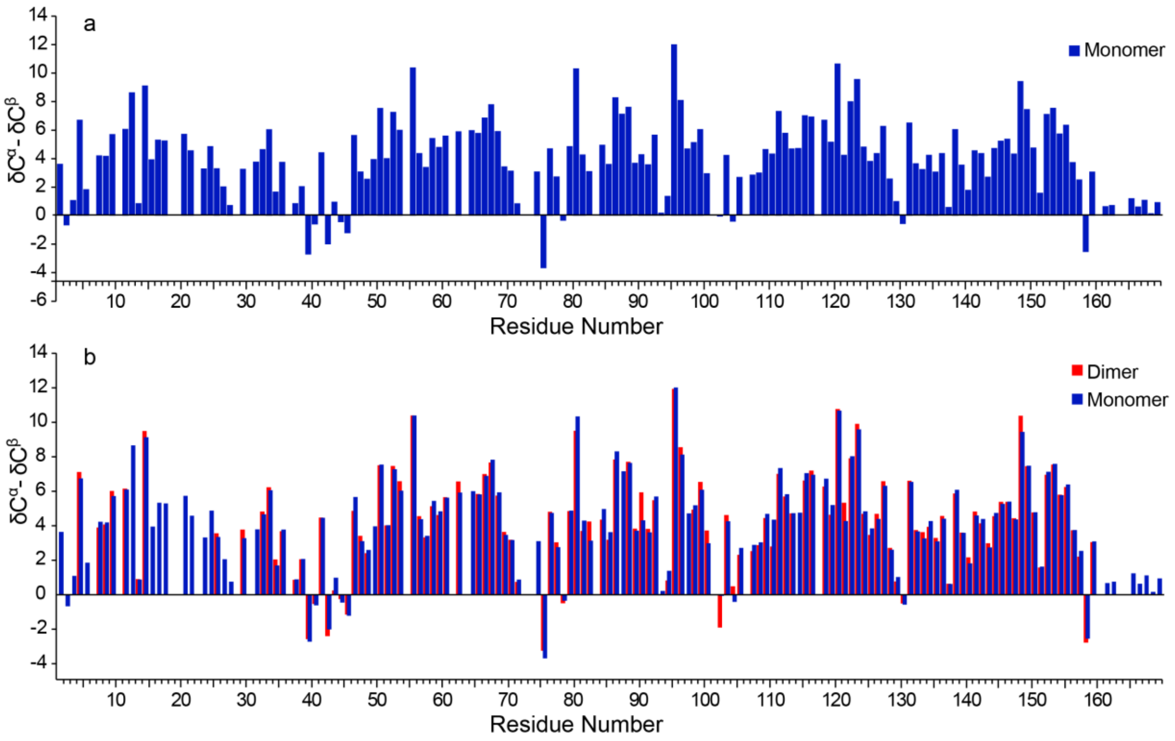

Supplementary Figure 5 | Secondary structure analysis of membrane-embedded G87V-mTSPO (blue) in complex with DAA1106. Positive δC^{α}-δC^{β} values are indicative of α-helix. Comparison with δC^{α}-δC^{β} values observed in wild-type mTSPO in complex with DAA1106 (red) demonstrate that the transmembrane helices are intact in both the monomeric and dimeric states of the protein.

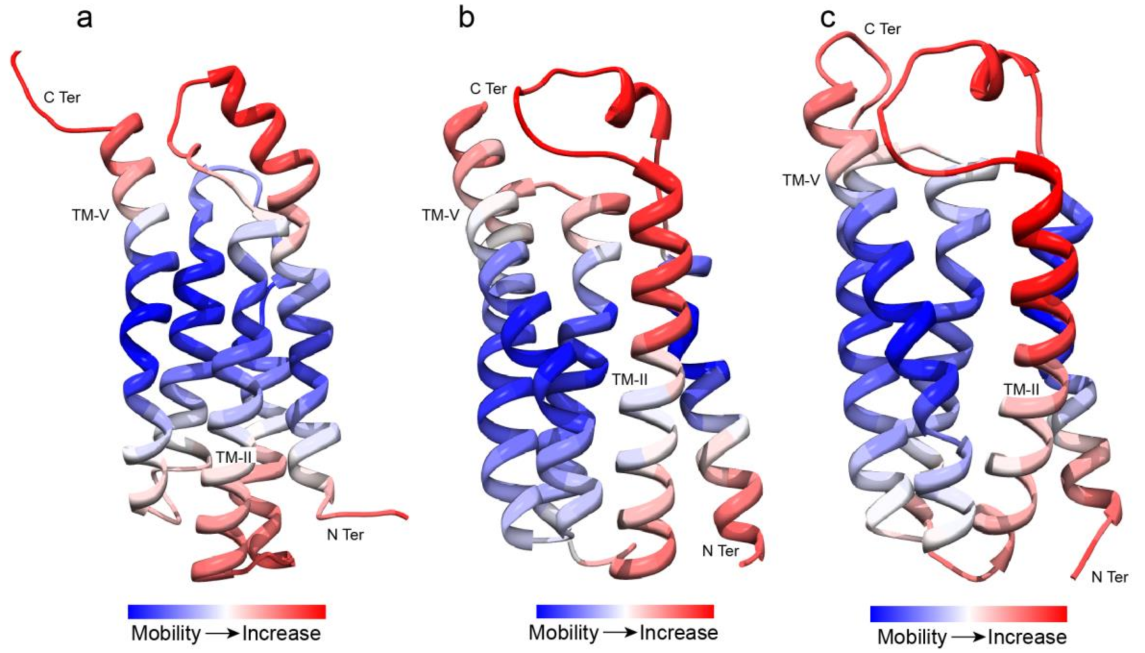

Supplementary Figure 6 | Gaussian network analysis applied to mTSPO and its bacterial homologues. (**a**) mTSPO (PDB id: 2MGY). (**b**) TspO from *Bacillus cereus* (PDB id: 4RYJ). (**c**) A138T-TspO from *Rhodobacter sphaeroides* (PDB id: 4UC2). The least stable transmembrane parts of the TSPO fold are the CRAC motif in TM-V and the transmembrane helix TM-II.

Supplementary Table 1 | Solid-state NMR experiments recorded for $^{13}C/^{15}N$-labelled mTSPO in complex with DAA1106. The mTSPO:DMPC molar ratio was 1:20.

Frequency (MHz)	Experiment	Acquisition details: Complex points; t_{max} (ms); SW	Scans	Spinning Speed (kHz)
950	2D NCA<br>$^{1}H/^{15}N$ Ramp CP (500µs)<br>$^{15}N/^{13}C$ Tang CP (3800 µs)	$\omega_1(^{13}C)$: 1792; 12.5; 299<br>$\omega_2(^{15}N)$: 80; 12.9; 32	144	19
950	2D NCO<br>$^{1}H/^{15}N$ Ramp CP (500µs)<br>$^{15}N/^{13}C$ Tang CP (4200 µs)	$\omega_1(^{13}C)$: 1792; 12.5; 299<br>$\omega_2(^{15}N)$: 80; 12.9; 32	160	19
950	3D NCACB<br>(CA-CB DREAM mixing [1])	$\omega_1(^{13}C)$: 1500; 10.5; 299<br>$\omega_2(^{13}C)$: 72; 5; 30<br>$\omega_3(^{15}N)$: 36; 5.9; 32	96	19
950	2D PDSD<br>$^{1}H/^{13}C$ Ramp CP (500µs)<br>20 ms mixing	$\omega_1(^{13}C)$: 1880; 15; 262<br>$\omega_2(^{13}C)$: 1260; 12; 220	112	11
950	3D NCACX<br>25 ms PDSD mixing	$\omega_1(^{13}C)$: 1792; 12.5; 299<br>$\omega_2(^{13}C)$: 80; 5.6; 30<br>$\omega_3(^{15}N)$: 32; 5.2; 32	96	11
950	3D NCACX<br>100 ms PDSD mixing	$\omega_1(^{13}C)$: 1792; 12.5; 299<br>$\omega_2(^{13}C)$: 72; 5; 30<br>$\omega_3(^{15}N)$: 36; 5.9; 32	80	11
950	3D NCOCX<br>40 ms DARR mixing [2,3]<br>100 ms DARR mixing	$\omega_1(^{13}C)$: 1792; 12.5<br>$\omega_2(^{13}C)$: 40; 5.6<br>$\omega_3(^{15}N)$: 32; 5.2	88<br>104	11
950	3D NCOCA<br>CO-CA BSH transfer [4]	$\omega_1(^{13}C)$: 1792; 12.5; 299<br>$\omega_2(^{13}C)$: 36; 5.8; 13<br>$\omega_3(^{15}N)$: 36; 5.9; 32	128	19
850	3D NCACO<br>CA-CO BSH transfer	$\omega_1(^{13}C)$:1200; 12.8; 220<br>$\omega_2(^{13}C)$:64; 5; 30<br>$\omega_3(^{15}N)$: 36; 6.5; 32	80	20
850	3D CANCO	$\omega_1(^{13}C)$:1200; 12.8; 233<br>$\omega_2(^{13}C)$:64; 5; 32<br>$\omega_3(^{15}N)$: 36; 6.5; 32	112	20

Supplementary Table 2 | Acquisition parameters of additional NMR experiments recorded for different ^{13}C/^{15}N-labelled mTSPO samples.

Frequency (MHz)	Experiment	Acquisition details: Complex points; t_{max} (ms); SW	Scans	Spinning Speed (kHz)
G87V-mTSPO/DAA1106:DMPC (1:20)				
950	2D NCA<br>$^{1}H/^{15}N$ Ramp CP (500μs)<br>$^{15}N/^{13}C$ Tang CP (3800 μs)	$\omega_1(^{13}C)$: 1792; 12.5; 299<br>$\omega_2(^{15}N)$: 80; 12.9; 32	256	19
950	2D NCO<br>$^{1}H/^{15}N$ Ramp CP (500μs)<br>$^{15}N/^{13}C$ Tang CP (3400 μs)	$\omega_1(^{13}C)$: 1792; 12.5; 299<br>$\omega_2(^{15}N)$: 80; 12.9; 32	160	19
850	3D NCACB<br>(CA-CB DREAM mixing)	$\omega_1(^{13}C)$: 1242; 10; 292<br>$\omega_2(^{13}C)$: 68; 5.3; 30<br>$\omega_3(^{15}N)$: 30; 5.8; 30	88	20
950	2D PDSD<br>$^{1}H/^{13}C$ Ramp CP (500μs)<br>20ms mixing	$\omega_1(^{13}C)$: 1880; 15; 262<br>$\omega_2(^{13}C)$: 1260; 12; 220	96	11
mTSPO/DAA1106:DMPC (1:80)				
950	2D NCA<br>$^{1}H/^{15}N$ Ramp CP (400μs)<br>$^{15}N/^{13}C$ Tang CP (3500 μs)	$\omega_1(^{13}C)$: 1792; 12.5; 299<br>$\omega_2(^{15}N)$: 80; 12.9; 32	848	19
950	2D PDSD<br>$^{1}H/^{13}C$ Ramp CP (400μs)<br>20 ms mixing	$\omega_1(^{13}C)$: 1880; 15; 262<br>$\omega_2(^{13}C)$: 1260; 12; 220	112	11
mTSPO:DMPC (1:50)				
950	2D NCA<br>$^{1}H/^{15}N$ Ramp CP (400μs)<br>$^{15}N/^{13}C$ Tang CP (4200 μs)	$\omega_1(^{13}C)$: 1792; 12.5; 299<br>$\omega_2(^{15}N)$: 88; 13.4; 34	320	19
950	2D NCO<br>$^{1}H/^{15}N$ Ramp CP (400μs)<br>$^{15}N/^{13}C$ Tang CP (4200 μs)	$\omega_1(^{13}C)$: 1792; 12.5; 299<br>$\omega_2(^{15}N)$: 88; 13.4; 34	320	19
950	2D PDSD<br>$^{1}H/^{13}C$ Ramp CP (400μs)<br>20ms mixing	$\omega_1(^{13}C)$: 1880; 15; 262<br>$\omega_2(^{13}C)$: 1260; 12; 220	104	11
mTSPO:DMPC (1:20)				
800	2D NCA<br>$^{1}H/^{15}N$ Ramp CP (800μs)<br>$^{15}N/^{13}C$ Ramp CP (3000 μs)	$\omega_1(^{13}C)$: 1184; 10; 296<br>$\omega_2(^{15}N)$: 92; 14.2; 40	304	11

800	2D NCO<br>$^{1}H/^{15}N$ Ramp CP (800μs)<br>$^{15}N/^{13}C$ Ramp CP (3800 μs)	$\omega_1(^{13}C)$: 1660; 14; 296<br>$\omega_2(^{15}N)$: 84; 14.4; 36	300	11
800	2D PDSD<br>$^{1}H/^{13}C$ Ramp CP (600μs)<br>20 ms mixing	$\omega_1(^{13}C)$: 1880; 15; 262<br>$\omega_2(^{13}C)$: 1260; 12; 220	128	11
Y152S-mTSPO/DAA1106:DMPC (1:20) / Y152S-mTSPO/DAA1106:DMPC:Chol (1:20:10)				
950	2D NCA<br>$^{1}H/^{15}N$ Ramp CP (500μs)<br>$^{15}N/^{13}C$ Tang CP (3800 μs)	$\omega_1(^{13}C)$: 1792; 12.5; 299<br>$\omega_2(^{15}N)$: 88; 13.4; 34	640/640	19
950	2D NCO<br>$^{1}H/^{15}N$ Ramp CP (500μs)<br>$^{15}N/^{13}C$ Tang CP (4200 μs)	$\omega_1(^{13}C)$: 1792; 12.5; 299<br>$\omega_2(^{15}N)$: 88; 13.4; 34	384/384	19
950	2D PDSD<br>$^{1}H/^{13}C$ Ramp CP (500μs)<br>20 ms mixing	$\omega_1(^{13}C)$: 1880; 15; 262<br>$\omega_2(^{13}C)$: 1260; 12; 220	96/120	11
mTSPO/DAA1106:DMPC:Cholesterol (1:20:10)				
950	2D NCA<br>$^{1}H/^{15}N$ Ramp CP (500μs)<br>$^{15}N/^{13}C$ Tang CP (3800 μs)	$\omega_1(^{13}C)$: 1792; 12.5; 299<br>$\omega_2(^{15}N)$: 88; 13.4; 34	144	19
950	2D NCO<br>$^{1}H/^{15}N$ Ramp CP (500μs)<br>$^{15}N/^{13}C$ Tang CP (4200 μs)	$\omega_1(^{13}C)$: 1792; 12.5; 299<br>$\omega_2(^{15}N)$: 88; 13.4; 34	160	19
950	2D PDSD<br>$^{1}H/^{13}C$ Ramp CP (500μs)<br>20 ms mixing	$\omega_1(^{13}C)$: 1880; 15; 262<br>$\omega_2(^{13}C)$: 1260; 12; 220	128	11

Supplementary methods

Synthesis of DAA1106. *N*-(2,5-Dimethoxybenzyl)-*N*-(5-fluoro-2-phenoxyphenyl)acetamide **5** (DAA1106) was prepared according to scheme 1 below [5]. 2,5-Dimethoxybenzyl **7** chloride was synthesized from 2,5-dimethoxybenzyl alcohol [6].

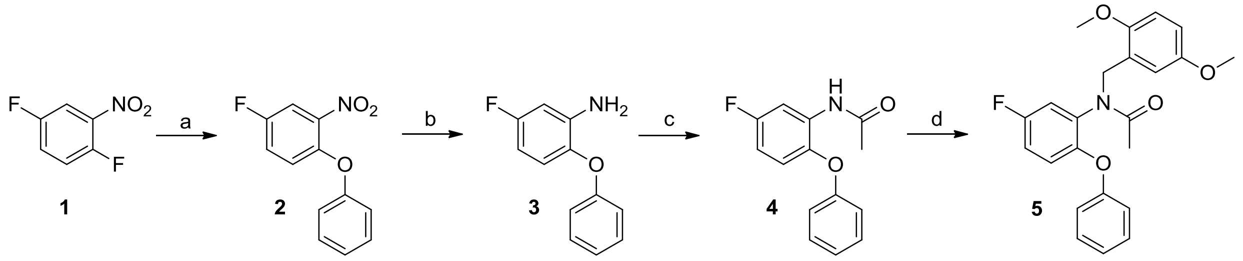

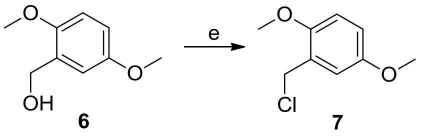

Reagents and conditions: (a) PhOH, K_2CO_3, DMF, 75 °C, 3 h, 52%; (b) Fe, AcOH, 100 °C, 30 min, 93%; (c) AcCl, *N*-methylmorpholine, CH_2Cl_2, 0-20 °C, 2 h, 97%; (d) 2,5-dimethoxybenzyl chloride, NaH, DMF, 2 h, 81%; (e) HCl conc., 20 min, 80%.

Supplementary References

1. Verel, R., Ernst, M. & Meier, B.H. Adiabatic dipolar recoupling in solid-state NMR: the DREAM scheme. *J Magn Reson* **150**, 81-99 (2001).
2. Morcombe, C.R., Gaponenko, V., Byrd, R.A. & Zilm, K.W. Diluting abundant spins by isotope edited radio frequency field assisted diffusion. *J Am Chem Soc* **126**, 7196-7 (2004).
3. Takegoshi, K., Nakamura, S. & Terao, T. C-13-H-1 dipolar-assisted rotational resonance in magic-angle spinning NMR. *Chem Phys Lett* **344**, 631-637 (2001).
4. Shi, C. et al. BSH-CP based 3D solid-state NMR experiments for protein resonance assignment. *J Biomol NMR* **59**, 15-22 (2014).
5. Okubo, T., Yoshikawa, R., Chaki, S., Okuyama, S. & Nakazato, A. Design, synthesis and structure-affinity relationships of aryloxyanilide derivatives as novel peripheral benzodiazepine receptor ligands. *Bioorg Med Chem* **12**, 423-38 (2004).
6. Mandell, L., Cooper, S.M., Rubin, B., Campana, C.F. & Day, R.A. Synthesis and electrochemical reduction of [2-(1,4-benzoquinonyl)ethyl]-1,4-benzoquinone. *The Journal of Organic Chemistry* **48**, 3132-3134 (1983).
